# Do-Not-ResuscitateDecision-Making during the COVID-19 Pandemic in a Teaching Hospital: Lessons Learned for the Future

**DOI:** 10.1155/2023/2771149

**Published:** 2023-12-20

**Authors:** Mick van de Wiel, Sabrina van Ierssel, Walter Verbrugghe, Veerle Mertens, Annelies Janssens

**Affiliations:** ^1^Antwerp University Hospital (UZA), Department of Thoracic Oncology, Drie Eikenstraat 655, Edegem 2650, Belgium; ^2^Antwerp University Hospital (UZA), Department of General Internal Medicine, Infectious Diseases and Tropical Medicine, Edegem 2650, Belgium; ^3^Antwerp University Hospital (UZA), Department of Intensive Care, Edegem 2650, Belgium; ^4^Antwerp University Hospital (UZA), Department of Geriatrics, Edegem 2650, Belgium

## Abstract

**Method:**

A cross-sectional survey study was conducted between February 2021 and April 2021 for all doctors and doctors in training, working in the Antwerp University Hospital during the COVID-19 pandemic.

**Results:**

127 doctors participated in this study. The familiarity with the different scores used in the triage during the COVID-10 pandemic was 51% for the Clinical Frailty Scale (CFS) and 20% for the Charlson Comorbidity Index (CCI). Participants indicated that their DNR decision is based on various aspects such as clinical assessment, comorbidities, patient's wishes, age, prognosis, and functional state.

**Conclusion:**

The familiarity with the different scores used during triage assessments is low. The total clinical picture of the patient is needed to make a considered decision, and this total picture of the patient seems to be well encompassed by frailty measurement (CFS). Although many participants indicated that the different scores do not offer much added value compared to their clinical assessment, it can help guide DNR decisions, especially for doctors in training.

## 1. Introduction

The recent COVID-19 pandemic entailed a new reality, namely, a possible shortage of care capacity with, more specifically, an increased demand for intensive care beds. Patients of advanced age or with significant comorbidities have little chance of a good outcome after prolonged intubation and admission to intensive care [1, 2]. This is reflected in a legitimate concern for the potentially important ethical dilemma of who may or may not occupy which bed or intensive care bed. The need for clear decisions on advanced care planning and the establishment of therapy restrictions in the form of a “do-not-resuscitate” (DNR) code is underlined once again [3–5].

During the pandemic, there was an international search for objective tools to support DNR decision-making. The Clinical Frailty Scale (CFS) is a reliable tool used to estimate the risk of poor outcome in elderly people in different chronic settings (patients with liver cirrhosis, oncological patients, patients with a geriatric profile, etc.) [6–10]. The CFS includes a clinical assessment of the patient's daily functioning, divided into nine categories (from 1 “very fit” to 9 “terminally ill”) [11, 12]. In COVID-19 patients, a CFS equal to or greater than 4 is associated with poor outcome [13]. The Charlson Comorbidity Index (CCI) is a validated method for predicting a patient's 10-year survival based on the present comorbidities. Each comorbidity is given a score of 1 (such as previous myocardial infarction), 2 (such as hemiplegia), 3 (such as moderate or severe liver disease), or 6 (such as malignant tumours) [14]. In COVID-19 patients, a CCI equal to or greater than 3 is associated with poor outcome [15, 16]. The use of the CCI is widespread, especially in longitudinal studies, but has not been renewed since its creation in 1987 [17, 18].

During the COVID-19 pandemic, both the CFS and CCI were used worldwide to guide decisions on triage of patients to certain departments [19–23]. A triage guideline and team were set up to decide on the allocation of patients in the event of force majeure at the Antwerp University Hospital (UZA). In these allocation rules, the assignment of level of care (hospital admission, intensive care admission, and discharge to home) will be determined by three aspects: the estimated survival of the patient, the patient's wishes, and the availability of resources (beds). The estimation of individual patient survival was conducted by means of a 10-point checklist whereby a positive answer to one or more of the items would reduce the chances of survival. Until now, these allocation guidelines had not been necessary in Belgium.

Additionally, to support difficult decisions concerning therapeutic restrictions within the Antwerp University Hospital (UZA), a multidisciplinary meeting (MDO-COVID) was set up during the pandemic. The multidisciplinary consultations brought together doctors from different disciplines: intensive care, infectious diseases, pneumology, emergency medicine, etc.; some more familiar with COVID-19 than others. Doctors doing rounds on the COVID wards, specialists and residents, were asked to prepare these meetings. The CFS and CCI were herein suggested as supporting tools to estimate prognosis in COVID-19 patients. As such, the idea arose to investigate how, from a doctor's perspective, the decision-making process to determine a DNR code is implemented in the day-to-day clinical practice of all doctors within a tertiary teaching hospital. We looked at the familiarity with the use of the different scores, also used during the multidisciplinary meeting, as possible objective support for these difficult discussions. These scores were used to support the allocation guidelines.

## 2. Methods and Materials

After approval by the ethics committee, a survey was sent out to all doctors and doctors in training at the Antwerp University Hospital (UZA). After obtaining informed consent, the survey could be completed. The survey was sent out during the first quarter of 2021, which corresponded to the period between the second and third COVID-19 peaks in Belgium. Doctors at the hospital were contacted through various channels: via the weekly COVID newsletter, via personal emails, and via flyers in the wards. About 430 doctors (including assistant physicians) at UZA were contacted in this way. In Belgium, doctors in training or residents are doctors who have already obtained their master's degree in medicine and are completing their specialist training.

The survey was created by four doctors in our research team who work in different departments (intensive care, infectious diseases, and thoracic oncology). Our most important objectives were to find out who is familiar with the scores that are used (CFS and CCI), and who already uses these scores in clinical practice and if not, why not? In addition, we also wanted to find out what therapeutic decisions would be made using difficult cases that were similar to the ones discussed during the pandemic. The survey can be found in Appendix 1 and consists of three parts. The first part contains demographic data of the participant (age, gender, religion, and working department). The second part consists of seven questions assessing the extent to which a doctor is faced with DNR decisions in daily practice, as well as familiarity with the different scores (CFS and CCI) used in decision-making. We also surveyed the possible positive and negative points of the various scores used. The third part consists of three cases, as also presented during the multidisciplinary meeting, in which a therapeutic decision had to be made.

The online survey program “LimeSurvey” was used to collect the survey data. Data was exported to SPSS version 27, which was used for descriptive analysis. Where relevant, we conducted a statistical analysis by employing the Pearson chi-square test to evaluate whether the observed differences were statistically significant. Excel was used for the qualitative analysis of the text responses. In a first step, the text responses were labelled using open codes close to the participants' words. Notes and interpretations were added. In a next step, these open codes and text fragments were compared and categorised in overarching themes, while differences in interpretations were discussed in a joint session. Finally, an overview of the different themes was made.

## 3. Results

**Figure 1:**
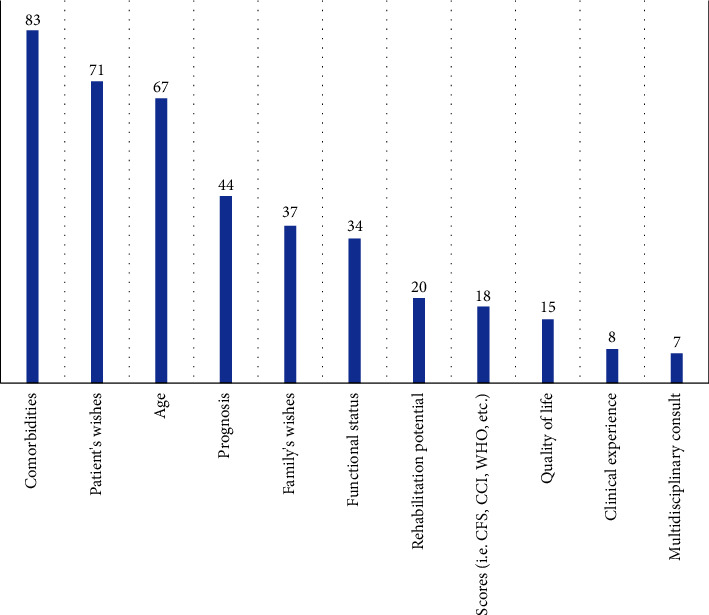
Most frequently cited responses to the question, “What do you base your decision-making on when determining a DNR code?”

A total of 161 of the 430 doctors (response rate 37%) contacted participated in this study. In 33 questionnaires, only demographic data were filled in and thus were not considered for further analysis.  Figure 1 shows the flow diagram and number of valid surveys for analysis (see Appendix 2).  Table 1 shows the demographics of the participants. One nurse filled in the questionnaire and was excluded from all further analysis.

Table 2 shows the individual involvement in DNR decision-making. In general, we see that most doctors indicate that they are involved in making DNR decisions on a weekly basis. Looking specifically at the difference between specialists and doctors in training, we see that the proportion of assistants involved in these decisions is higher compared to the number of specialists (37–40% of assistants compared to 25–30% of specialists). As indicated by the chi-square test, there was no statistically significant difference between both groups.

Table 3 shows the number of participants answering “yes” to the stated questions. We see that residents were more often assigned to a COVID ward than permanent specialist staff members (70% vs. 36%). When we look at participation in the multidisciplinary meetings, we see a lower percentage of residents compared to specialists (32% vs. 40%). When we compare the same situation for internal medicine versus surgical disciplines, we see that internists were more often assigned to a COVID department (69% vs. 32%) and as a consequence, internists also participated in the multidisciplinary meetings more often compared to surgeons (44% vs. 25%). As indicated by the chi-square test, there was no statistically significant difference between the groups regarding the multidisciplinary meeting, but there was a statistically significant difference between the groups regarding the assignment to a COVID department.

Table 4 shows the degree of familiarity with the scores used in the multidisciplinary meeting. Looking at the CFS, most doctors (51%) were familiar with this score from before the pandemic, 19% of doctors learned of the score during the pandemic, and 30% of doctors were unfamiliar with this score. From the text responses (Tables 5 and 6), we learned that most of the physicians knew this score through its use in scientific work, through geriatric screening and also since the COVID-19 pandemic. Mentioned positive points of this score are that it can provide a quick assessment of functional status, that it is perceived as useful in supporting a decision, and that it is also considered a rather reliable score. Negative points raised are the subjectivity of the score, the fact that anamnesis or heteroanamnesis is not always possible in an acute setting, and others see this score as rather unreliable with no added value compared to their clinical assessment.

The CCI is not as well known. Only 20% of the doctors were familiar with this score before the pandemic, 28% learned of the score during the pandemic, and 52% of the doctors say they are unfamiliar with this score. The score is mainly known from its use during the pandemic and from scientific work. Mentioned positive points are its reliability and usefulness as a decision aid. Negative points listed are that the score is rather outdated and not relevant to the present time. Also, the severity of a comorbidity is not always taken into consideration when quantifying the score.

Table 7 shows the therapeutic decisions that doctors would make in the respective cases presented in the survey (cases are also available in Appendix 1). All cases question what decision would be made in the event of respiratory deterioration due to COVID-19. Case 1 concerns a young man (33 years) with Steinert's disease and moderate functional status. In the event of respiratory deterioration, the majority of respondents (52%) opted for a transfer to intensive care with intubation if necessary. Case 2 concerns an elderly man (82 years) with few or no comorbidities and good functional status. In this case, the majority of respondents (51%) opted for a restrictive policy with only high flow nasal oxygen therapy (HFNO) in the ward if the patient's respiratory function deteriorated. Case 3 concerns a man (65 years) with many cardiac and respiratory comorbidities but relatively good functional status. Here too, the majority (50%) opted for a restrictive ward policy with HFNO only.

When we compare the decisions made in the survey with the decisions taken in real life, we see that they do not always correspond. In the first case, due to the severe mental retardation and already needing respiratory support in the home setting, a more restrictive policy was applied. In the second case, the decision was made to intubate the patient in case of deterioration. This decision was made primarily because of the patient's very low frailty scale. In the third case, the decision was initially made to apply a restrictive policy with HFNO only because of the many comorbidities. However, the patient presented with respiratory distress at a time when the pressure on beds was very low, and due to the patient's age, the decision was made to transfer the patient to intensive care and to intubate. Five months after intubation and after a long stay in intensive care, the patient was able to leave the hospital for further rehabilitation.

Table 8 shows possible issues that may influence the decision regarding therapeutic restrictions. For all three issues surveyed, namely, conducting the DNR interview oneself, wishes of family and the patient's religion, the most frequent answer was that this has no influence on the DNR decision-making (55%, 48%, and 52% respectively).

Figure 1 shows the text responses to the question, “What do you base your decision-making on when determining a DNR code?” The most frequently mentioned aspects are the patient's comorbidities, patient's wishes and patient's age. The least mentioned aspects are quality of life, clinical experience and multidisciplinary consult (discussion with colleagues). The text responses underscore the complexity of advanced care planning, which is not based on only one aspect, but is based on a combination of multiple criteria that do not weigh equally in each case. Respondents do mention a nuanced response when it comes to taking into account the wishes of the patient and their family. Namely that this mainly means not extending care when the patient themselves do not want an extension. However, when a patient wishes everything to be done and this is not realistic or feasible on medical grounds, many state that the clinical assessment (functional status and prognosis) is the deciding factor.

Table 9 shows the responses given to the question “Do you think the COVID-19 pandemic may influence the initiation of end-of-life conversations (i.e., DNR discussions)?” Most respondents (55%) indicated that the COVID-19 pandemic made the conversation easier, 40 respondents (25%) believed it made the conversation more difficult, and 33 respondents (20%) stated that they do not know whether the pandemic affected the conversation.

## 4. Discussion

The strain on healthcare systems during the COVID-19 pandemic brought attention to the need for adequate triage and bed allocation. An important part of this is establishing guidelines for therapeutic restrictions, of which the “do-not-resuscitate” (DNR) code is a globally known tool. Our survey shows that DNR codes are determined daily by both specialists and residents. Despite this fact, there is a paucity of data available in the literature regarding DNR decision-making and conducting end-of-life conversations.

In practice, doctors are not very familiar with the different scores used in the literature to guide DNR decisions and bed allocation. Only 50% of doctors said they were already familiar with the Clinical Frailty Scale (CFS) and Charlson Comorbidity Index (CCI) used to guide DNR decisions during the pandemic. As stated in the text responses of our survey, many physicians indicated that, during DNR decision-making, they do not consider the scores to offer any added value over their clinical view, experience, and personal assessment (or in other words their “gut feeling”) when assessing the patient's condition. Indeed, only a small number of physicians take these scores into account in their DNR decision-making.

### 4.1. Clinical Frailty Scale

In our survey, the Clinical Frailty Scale (CFS) was generally considered to be reliable but often difficult to assess without an anamnesis or a heteroanamnesis. Determining the CFS requires a review of the patient's complete functional picture with opinions from the patient, their family, attending physician and general practitioner, and therefore cannot only be deduced from an acute clinical setting. Furthermore, the responses in the study show that conducting a thorough anamnesis or heteroanamnesis in acute setting is considered difficult, as gathering this information is often time-consuming.

Several studies show that the CFS, which is primarily a description of a patient's functional status, is a better prediction of outcome than age or comorbidities, on which the Charlson Comorbidity Index (CCI) largely relies on [24, 25]. It therefore appears that the CFS is a more preferable decision aid in evaluating patients for possible therapeutic restrictions. As said before, the CFS forces one to look beyond a purely prognostic picture and objective parameters. Involvement of discipline-specific treating physicians, general practitioner, and heteroanamnesis via the family is of great added value in determining a CFS. Particularly in an acute setting, it is not always possible to assess the complete situation immediately. This in turn highlights the fact that an acute clinical setting where immediate live-saving interventions are required is not the ideal time to discuss a DNR code.

### 4.2. Charlson Comorbidity Index

The Charlson Comorbidity Index (CCI) is seen as reliable but outdated. It has not been revised since its creation in 1987, and the current prognosis for both cancer and HIV has changed tremendously since then [26, 27]. Another notable aspect is the fact that the severity of a comorbidity is not taken into account in the score (i.e., one point for COPD regardless of the classification).

### 4.3. DNR Conversations

Determining a DNR code is very difficult, not only from the doctors' perspective, but also from the patients' perspective. This makes initiating DNR conversations very challenging. Preferably, these conversations should be held earlier in a doctor's treatment relationship with a patient. As indicated by the majority of respondents, we believe that the COVID-19 pandemic can serve as a lead-in to initiate end-of-life conversations [28–30].

Students at our university experience a lack of confidence in the communication skills used in end-of-life conversations [31]. In this study, looking at everyday practice, we see that especially residents are confronted with determining DNR codes and conducting these conversations. This is not easy and as we indicated earlier, experience and a kind of “gut feeling” are also very important in this process, two aspects that are still in development in doctors at the start of their careers. The full clinical picture of the patient, with both subjective and objective parameters, is important to guide a decision regarding therapeutic restrictions.

However, the Clinical Frailty Scale (CFS) can be used by residents as an objective tool to guide their decision-making on DNR codes, as the CFS seems to be a good reflection of this “gut feeling” [32]. This can be an important message to give as a supervisor to residents. Regarding DNR conversations, it seems appropriate that residents are supported by specialists in how to conduct these conversations and convey clear messages to the patient and their family.

### 4.4. Multidisciplinary Consultations

Because of the need to have a clear DNR policy for each patient, multidisciplinary consultations on treatment restrictions were put in place during the COVID-19 epidemic waves. During these so-called MDO-COVID meetings, there was room and space to look at a patient's complete picture and to make a clear DNR code decision in consultation with treating physicians. Doctors from different disciplines and with different opinions were able to learn from each other in this way. When a new pathology presents itself (such as the COVID-19 pandemic), such consultations can be extremely useful, not only as support for difficult DNR decisions, but also because it accelerated the clinical learning curve. The results from our survey show that it was mainly internists who were assigned to the COVID departments and who participated in the multidisciplinary meetings. This was to be expected given the medical aspects of the condition. Residents participated in these multidisciplinary meetings less frequently, despite the fact that they were more often assigned to the COVID departments than permanent staff. Even outside the pandemic, residents, whether or not in consultation with colleagues or the patient's family, report being involved in DNR decision-making on a weekly basis.

Considering the cases presented in the survey, we see fairly uniform answers concerning therapeutic restrictions. The discussions in the multidisciplinary consultations clearly provided a more nuanced picture that is not encompassed by medical history and scores alone. Therefore, the decisions made in real life for these cases do not always correspond to the decisions made in the survey. Therefore, it seems useful for us to study such cases and engage in discussions as a group during medical training. In addition, approximately half of the physicians indicated that they were influenced by factors such as having to conduct the DNR conversation themselves, the wishes of the family, or the patient's religion. Although in theory, it seems easy to decide on a DNR code on paper or in a team, in practice, it is different when you have a therapeutic relationship with the patient (and their family). A family discussion or a discussion with the patient can be difficult and often leads to a different DNR code. During the pandemic, with the impending pressure on our healthcare system and the media attention that was given to it, it seemed easier to communicate to the patient and family that therapeutic restrictions were being applied. There seemed to be more understanding that patients with a better chance of good survival were more likely to receive an extension of care. The question is whether this more openly communication will remain after the pandemic.

### 4.5. Limitations

Limitations of our study are the fact that only doctors from one university centre were surveyed. Because of the low response rate, a possible bias is that more doctors with a preexisting interest in the subject filled in the survey, so we cannot draw conclusions for the entire group of doctors. The fact that some of the results are not statistically significant is also attributed to the small study population.

## 5. Conclusion

Determining DNR codes is part of the daily practice of doctors and even doctors in training. An overall picture of the patient is necessary to determine which interventions will more likely lead to beneficial outcomes and which may be more harmful to particular individuals. Doctors use their clinical view and experience, mainly based on a variety of patient criteria, to guide their DNR decision-making. This total picture seems to be well encompassed by frailty measurement. Although many physicians indicated that the scores used do not offer much added value compared to their clinical assessment, especially the Clinical Frailty Scale (CFS) can help give doctors in training an objective tool for making decisions about DNR codes. The Clinical Frailty Scale can be used as a guide for making decisions about DNR codes. When determining the CFS, it is necessary to question others (such as the patient's family and general practitioner). This again highlights the fact that end-of-life discussions should be held in consultation and not in an acute event.

## Figures and Tables

**Table 1:** Demographics (*n* = 128).

Gender	
Male	66 (52%)
Female	61 (47%)
Other identification	1 (1%)
Age	
Min–max (years)	24–65
Mean (years)	36
Religion	
None	65 (51%)
Catholic	54 (42%)
Islam	5 (4%)
Buddhism	1 (1%)
Hinduism	1 (1%)
Other	2 (2%)
Position	
Specialist	60 (47%)
Resident	67 (52%)
Nurse	1 (1%)
Specialty	
Internal medicine	87 (68%)
Surgical disciplines	28 (22%)
Technical disciplines	9 (6%)
Not specified	4 (3%)

**Table 2:** Involvement in DNR decision-making (Sp: specialist; R: resident) (*n* = 127).

How often are you involved in the DNR decision (independent of the COVID-19 pandemic)?	Almost daily		Weekly		Monthly		Less than monthly		Never	
	Sp	R	Sp	R	Sp	R	Sp	R	Sp	R
DNR decision without contact with patient	12 (20%)	9 (13%)	14 (23%)	25 (37%)	8 (13%)	6 (9%)	11 (18%)	16 (24%)	15 (25%)	11 (16%)
	*X* ^2^ (4, 127) = 4.987, *p*=0.289									

DNR decision after consultation with patient and/or their family	6 (10%)	10 (15%)	15 (25%)	26 (39%)	13 (22%)	14 (21%)	14 (23%)	11 (16%)	12 (20%)	6 (9%)
	*X* ^2^ (4, 127) = 5.981, *p*=0.201									

DNR decision in consultation with colleagues	6 (10%)	13 (19%)	19 (32%)	27 (40%)	12 (20%)	8 (12%)	14 (23%)	13 (19%)	9 (15%)	6 (9%)
	*X* ^2^ (4, 127) = 5.037, *p*=0.284									

**Table 3:** Number of participants (percentage) answering “yes” to the following questions (*n* = 127).

	Position		Specialty		
	Specialist (*n* = 60)	Resident (*n* = 67)	Internal medicine (*n* = 90)	Surgical disciplines (*n* = 28)	Technical disciplines (*n* = 9)
Were you assigned to a COVID department during the pandemic?	22 (36%)	47 (70%)	60 (67%)	9 (32%)	0 (0%)
	*X* ^2^ (1,127) = 14.302, *p* < 0.001		*X* ^2^ (2,127) = 21.782, *p* < 0.001		

Did you participate in the multidisciplinary meeting during the pandemic?	24 (40%)	22 (32%)	38 (42%)	7 (25%)	1 (11%)
	*X* ^2^ (1,127) = 0.703, *p*=0.401		*X* ^2^ (2,127) = 5.386, *p*=0.067		

**Table 4:** Degree of familiarity with the prognostic scores (*n* = 127).

	Yes, from before the pandemic	Yes, since the pandemic	No
Are you familiar with the use of the Clinical Frailty Scale (CFS)?	64 (50%)	24 (19%)	39 (31%)
Are you familiar with the use of the Charlson comorbidity index (CCI)?	25 (20%)	36 (28%)	66 (52%)

**Table 5:** Qualitative codes extracted from participants' responses in the descending order of the frequency that it was mentioned (times mentioned between brackets) by the participants regarding the use of the Clinical Frailty Scale (CFS).

Familiarity with the use of the score via
Geriatrics [24]
Clinical studies [6]
COVID ward [5]
Intensive care unit [4]
Preoperative assessment [2]
Oncology department [2]
Emergency department [1]
Positive aspects of the score
Reliable [12]
Useful and guiding in DNR decision-making [10]
Applicable to use in clinic [5]
Quick assessment [2]
Negative aspects of the score
Arbitrary/not based on objective questions [5]
Limited reproducibility between colleagues/subjective [3]
Limited value in acute setting [1]
Clinical assessment is equally reliable [1]
Unreliable [1]
Anamnesis or heteroanamnesis required for proper evaluation [1]

**Table 6:** Qualitative codes extracted from participants' responses in descending order of the frequency (times mentioned between brackets) that it was mentioned by the participants regarding the use of the Charlson Comorbidity Index (CCI).

Familiarity with the use of the score via
COVID ward [8]
Clinical studies [7]
Intensive care unit [4]
Geriatrics [2]
Emergency department [2]
Oncology [2]
Preoperative assessment [1]
Positive aspects of the score
Useful and guiding in DNR decision-making [8]
Reliable [6]
Objective [4]
Representative [3]
Negative aspects of the score
Poor representation of current clinical condition/limited reliability for short-term relevance [6]
Severity of comorbidity is not always taken into consideration [4]
Outdated/irrelevant for current practice [3]
Difficult to handle [3]

**Table 7:** Therapeutic decisions on cases as presented during the pandemic HFNO: high flow nasal oxygen.

	Maximum care on ward	High flow nasal oxygen therapy (HFNO) on the ward	Transfer to intensive care unit with intubation if required (thus no therapy restrictions)	I don't know/other	Real-life clinical decision
Case 1 (*n* = 122)	13 (11%)	38 (31%)	65 (53%)	6 (5%)	HFNO on the ward
Case 2 (*n* = 119)	6 (5%)	65 (55%)	43 (36%)	5 (4%)	Transfer to intensive care unit with intubation if required
Case 3 (*n* = 119)	42 (35%)	64 (53%)	10 (8%)	3 (3%)	HFNO on the ward

**Table 8:** Possible influences on therapeutic decisions (*n* = 127).

Would your decision be influenced if…	Yes	No	I don't know	Invalid response
… you have to conduct the DNR conversation yourself?	30 (24%)	69 (54%)	23 (18%)	5 (4%)
… the family wishes there to be no therapeutic restrictions?	36 (28%)	61 (48%)	22 (17%)	8 (6%)
… the patient states that their religion does not permit therapeutic restrictions?	20 (16%)	67 (53%)	32 (25%)	8 (6%)

**Table 9:** Influence of the COVID-19 pandemic on end-of-life conversations (*n* = 127).

The COVID-19 pandemic makes (initiating) the end-of-life conversation (i.e.; DNR conversations)…	
… easier	88 (55%)
… more difficult	39 (25%)

## Data Availability

The data that support the findings of this study are available from the corresponding author upon reasonable request.

## Abbreviations

CCI: Charlson Comorbidity Index
CFS: Clinical Frailty Scale
COPD: Chronic obstructive pulmonary disease
COVID: Coronavirus disease
DNR: Do not resuscitate
HFNO: High flow nasal oxygen
HIV: Humane immunodeficiency virus.

## Additional Points

*Article Summary (Strengths and Limitations of This Study)*. (i) This study contributes to the understanding of DNR decision-making and conducting end-of-life conversations, about which there is a paucity of data available in the current literature. (ii) This study maps the heterogeneous aspect of a DNR decision with all its various facets and does not reduce it to a single score that can be used to support decisions at a glance. (iii) Frailty measurement provides a good insight into a patient's functional status and can be used as a guide to help starting doctors make judgements about possible therapeutic restrictions. (iv) Doctors from only one university centre were surveyed with a low response rate, creating a possible bias to doctors with a preexisting interest in the surveyed subject.

## Supplementary Materials

Supplementary Materials Appendix 1: Survey. Example of the survey that was used for data collection. The survey consists of three parts. The first part contains demographic data of the participant (age, gender, religion, working department). The second part consists of seven questions assessing the extent to which a doctor is faced with DNR decisions in daily practice, as well as familiarity with the different scores (CFS and CCI) used in decision-making. We also surveyed the possible positive and negative points of the various scores used. The third part consists of three cases, as also presented during the multidisciplinary meeting, in which a therapeutic decision had to be made. Appendix 2: Flow diagram. The flow diagram shows the number of valid surveys used for analysis.
