# Supplementary material for: Do-Not-ResuscitateDecision-Making during the COVID-19 Pandemic in a Teaching Hospital: Lessons Learned for the Future
Source: J Aging Res. 2023 Dec 20;2023:2771149. doi: 10.1155/2023/2771149 (PMC10752667; doi:10.1155/2023/2771149)
Supplement: Supplementary Materials — Appendix 1: Survey. Example of the survey that was used for data collection. The survey consists of three parts. The first part contains demographic data of the participant (age, gender, religion, working department). The second part consists of seven questions assessing the extent to which a doctor is faced with DNR decisions in daily practice, as well as familiarity with the different scores (CFS and CCI) used in decision-making. We also surveyed the possible positive and negative points of the various scores used. The third part consists of three cases, as also presented during the multidisciplinary meeting, in which a therapeutic decision had to be made. Appendix 2: Flow diagram. The flow diagram shows the number of valid surveys used for analysis. [file 2771149.f1.zip › Appendix 1 - Survey.docx]

Appendix 1: Survey

# Demographics

| **Age** | _ _ years |
| --- | --- |
| **Gender** | ☐ Male  ☐ Female  ☐ Other identification  ☐ I do not wish to answer |
| **To which religious belief do you belong?** | ☐ None  ☐ Buddhism  ☐ Christianity  ☐ Hinduism  ☐ Islam  ☐ Judaism  ☐ Other: Please specify _____________________ |
| **In which specialty are you working?** | ☐ Specialist: _________________________  ☐ Resident:_________________________  ☐ Other: Please specify________________________ |
| **Were you assigned to a COVID department (or Red Zone emergency room) during the pandemic?**  ☐ Yes ☐ No | |
| **Did you participate in the MDO-COVID consultation during the pandemic?**  ☐ Yes ☐ No | |
| **Are you familiar with the use of the Charlson Comorbidity Index?**  ☐ Yes, from before the COVID-19 pandemic  ☐ Yes, since the COVID-19 pandemic  ☐ No  **If so, what is your experience with using the Charlson Comorbidity Index? (In what context did you come across this score or do you use this score? Do you find this score reliable?)**  ________________________________________________________________________________________________________________________________________________________________________________________________________________________________________________ | |
| **Are you familiar with the use of the Clinical Frailty Scale?**  ☐ Yes, from before the COVID-19 pandemic  ☐ Yes, since the COVID-19 pandemic  ☐ No  **If so, what is your experience with using the Clinical Frailty Scale? (In what context did you come across this score or do you use this score? Do you find this score reliable?)**  ________________________________________________________________________________________________________________________________________________________________________________________________________________________________________________ | |
| **How often are you involved in the DNR decision (independent of the COVID-19 pandemic)?**   \|  \| Almost daily \| Weekly \| Monthly \| Rare \| Never \| \| --- \| --- \| --- \| --- \| --- \| --- \| \| DNR decision **without** contact with patient (in a multidisciplinary meeting) \|  \|  \|  \|  \|  \| \| DNR decision **after consultation** with patient and/or their family \|  \|  \|  \|  \|  \| \| DNR decision in consultation **with colleagues** \|  \|  \|  \|  \|  \| | |
| **What do you base your determination of a DNR code on (independent of the COVID-19 pandemic) (e.g. patient age, comorbidities, patient/family preferences, certain scores, etc.)?** | |
| **Do you think the COVID-19 pandemic may influence the initiation of end-of-life conversations (i.e. DNR discussions)?**  ☐ It makes the conversation easier.  ☐ It makes the conversation more difficult. | |

## CASE 1

| Male; 33 years  **Past history:**   - Palatoschisis - Scoliosis - Mental retardation (6-year old level) - Non-ADL independent in view of cognitive impairment - Steinert’s disease (Myotonic Dystrophy) for which BiPAP since August 2020   Social: Patient lives with his older sister who takes care of patient on a daily basis. Patient is reasonably active, has a mobility of about 1 km and occasionally helps in the hair salon. |
| --- |
| - Charlson Comorbidity Index: 0 (if limited cognition counts; 1) - Clinical Frailty Score: 5 (mild frailty) |
| *For your information*  The Charlson Comorbidity Index (CCI) predicts 10-year survival in patients with multiple comorbidities. The higher the number, the lower the 10-year survival. **A CCI of 0 corresponds to a 10-year survival rate of 98%.**  The Clinical Frailty Scale (CFS) is a tool based on a clinical assessment of the patient in multiple areas. The higher the number, the higher the frailty. **A CFS of 5 corresponds to mild frailty (=activities are limited or delayed, more complex tasks require third-party assistance).** |

**Which statement do you feel applies to this patient in case of respiratory deterioration is due to COVID-19? There is no other organ failure.**

☐ Maximum care on ward (with own BiPAP)

☐ High flow nasal oxygen therapy in the ward (HFNO unit)

☐ Transfer to intensive care unit with intubation if required

☐ Other (please specify in text field)

**Would this decision be influenced if you had to conduct the DNR interview with the patient yourself?**

☐ Yes

☐ No

☐ I don’t know

## CASE 2

| Male, 82 years  **Past history:**   - Carpal Tunnel Release right - 2016: CVA (fully recovered) - Arterial Hypertension - Intervention in the back - 2018: Subdural hematoma after fall   Today: Via ambulance to emergency room, difficult anamnesis in view of confusion.  Hetero anamnesis via wife: fell on bath tub edge last week and since then permanent pain on left ribcage. |
| --- |
| - Charlson Comorbidity Index: 5 - Clinical Frailty Score: 3 (self-reliant) |
| *For your information*  The Charlson Comorbidity Index (CCI) predicts 10-year survival in patients with multiple comorbidities. The higher the number, the lower the 10-year survival. **A CCI of 5 corresponds to a 10-year survival rate of 21%.**  The Clinical Frailty Scale (CFS) is a tool based on a clinical assessment of the patient in multiple areas. The higher the number, the higher the frailty. **A CFS of 3 corresponds to “managing well” (=medical problems are well controlled, but these people are not regularly active beyond routine walking).** |

**Which statement do you consider applicable in this patient in case of respiratory deterioration due to COVID-19? Aside from the respiratory problems, a positive FOB is documented.**

☐ Maximum care in the ward

☐ High flow nasal oxygen therapy in the ward (HFNO unit)

☐ Transfer to intensive care unit with intubation if required

☐ Other (please state in text field)

**The patient’s family calls you later in the day and does not wish to restrict therapy. Would this influence your decision?**

☐ Yes

☐ No

☐ I don’t know

## CASE 3

| Male; 65 years  **Past history:**   - 1995: Diabetes Mellitus Type II; diabetic nephropathy - 04/2002: CABG with LIMA-LAD (OPCAB) - 2003: Retinopathy - 2006: Axonal polyneuropathy - 2006: Depression - 12/2008: Coronary stenting (drug eluting?) - 12/2008: Acute renal insufficiency (prerenal) - 01/10/2019: Attempted desobstruction from a subocclusive lesion Circumflex (unsuccessful), PCI D1 with POBA - 19/2/2019 and 24/01/2020: Acute pulmonary oedema with underlying ischaemic cardiomyopathy - 24/01/2020: Cardiorenal dilemma - 20/12/2019: Liver Cirrhosis - 06/2020: Chronic renal insufficiency stage IIIb/IV due to diabetic nephropathy and factor nephroangiosclerosis (tentative diagnosis) - 06/2020: Admission due to acute hyperkalemia in acute on chronic renal insufficiency and heart failure therapy |
| --- |
| - Charlson Comorbidity Index: 9 - Clinical Frailty Score: 4 (pre-frail) |
| *For your information*  The Charlson Comorbidity Index (CCI) predicts 10-year survival in patients with multiple comorbidities. The higher the number, the lower the 10-year survival. **A CCI of 9 corresponds to a 10-year survival rate of 0%.**  The Clinical Frailty Scale (CFS) is a tool based on a clinical assessment of the patient in multiple areas. The higher the number, the higher the frailty. **A CFS of 4 corresponds to “vulnerable” (= not dependent on others for daily help, but symptoms limit activities)** |

**Which statement would you apply to this patient in case of respiratory deterioration due to COVID-19? In addition to the respiratory problems, there is known cardiorenal insufficiency.**

☐ Maximum care in the ward

☐ High flow nasal oxygen therapy in the ward (HFNO unit)

☐ Transfer to intensive care unit with intubation if required

☐ Other (please state in text field)

**The patient states that due to his religious background he does not wish to restrict therapy. Would this influence your decision?**

☐ Yes

☐ No

☐ I don’t know
