# Supplementary material for: Do-Not-ResuscitateDecision-Making during the COVID-19 Pandemic in a Teaching Hospital: Lessons Learned for the Future
Source: J Aging Res. 2023 Dec 20;2023:2771149. doi: 10.1155/2023/2771149 (PMC10752667; doi:10.1155/2023/2771149)
Supplement: Supplementary Materials — Appendix 1: Survey. Example of the survey that was used for data collection. The survey consists of three parts. The first part contains demographic data of the participant (age, gender, religion, working department). The second part consists of seven questions assessing the extent to which a doctor is faced with DNR decisions in daily practice, as well as familiarity with the different scores (CFS and CCI) used in decision-making. We also surveyed the possible positive and negative points of the various scores used. The third part consists of three cases, as also presented during the multidisciplinary meeting, in which a therapeutic decision had to be made. Appendix 2: Flow diagram. The flow diagram shows the number of valid surveys used for analysis. [file 2771149.f1.zip › Supplementary material files - description.docx]

**Supplementary material files: short description**

Appendix 1: Survey

Example of the survey that was used for data collection. The survey consists of three parts. The first part contains demographic data of the participant (age, gender, religion, working department). The second part consists of seven questions assessing the extent to which a doctor is faced with DNR decisions in daily practice, as well as familiarity with the different scores (CFS and CCI) used in decision-making. We also surveyed the possible positive and negative points of the various scores used. The third part consists of three cases, as also presented during the multidisciplinary meeting, in which a therapeutic decision had to be made.

Appendix 2: Flow diagram

The flow diagram shows the number of valid surveys used for analysis.
